# Lotus Seeds: Current Molecular Biology Insights and Future Perspectives as a Prominent Biological Resource

**DOI:** 10.3390/plants15010136

**Published:** 2026-01-02

**Authors:** Jia Xin, Ruirui Li, Juan Liu, Xianbao Deng, Dong Yang, Heyun Song, Minghua Zhang, Hui Yang, Runjie He, Yapei Zhang, Heng Sun, Mei Yang

**Affiliations:** 1 State Key Laboratory of Plant Diversity and Specialty Crops, Wuhan Botanical Garden, Chinese Academy of Sciences, Wuhan 430074, China; xinjia21@mails.ucas.ac.cn (J.X.);; 2 University of Chinese Academy of Sciences, Beijing 100049, China; 3 College of Life Sciences, Xinyang Normal University, Xinyang 464000, China

**Keywords:** lotus seed, molecular biology, biosynthetic mechanism, postharvest, longevity

## Abstract

Lotus (*Nelumbo nucifera* Gaertn.) is an economically and medicinally significant aquatic plant, with its seeds (lotus seeds) having attracted considerable attention due to their unique developmental traits and abundance of bioactive and nutritional components. Over recent decades, advancements in lotus genome annotation and assembly have facilitated comprehensive investigations into the molecular biology of lotus seeds. Key genes involved in the biosynthesis of nutrients and bioactive compounds within lotus seeds have now been identified and functionally validated. This review comprehensively summarizes the latest advancements in the molecular biology of the edible and medicinal properties of lotus seeds, focusing on the biosynthetic mechanisms of key nutrients, such as starch, flavonoids, and alkaloids, as well as the molecular mechanisms underlying lotus seed developmental processes. Additionally, we present a detailed overview of the mechanisms involved in the postharvest preservation of fresh lotus seeds and their exceptional longevity. Based on the current progress in lotus seed molecular biology, we propose future research directions and methodologies. This review not only deepens the understanding of the molecular biology of lotus seeds but also provides valuable theoretical insights and practical guidance for promoting the genetic improvement and sustainable development of the lotus seed industry.

## 1. Introduction

Lotus is a perennial aquatic plant belonging to the genus *Nelumbo* in the family Nelumbonaceae, comprising two extant species: the Asian lotus (*Nelumbo nucifera* Gaertn.) and the American lotus (*Nelumbo lutea* Pers.) [1,2]. These species exhibit notable differences in floral morphology, flower color, and plant architecture [2]. The Asian lotus is extensively cultivated across Asia, characterized by tall stature, intricate floral morphologies, diverse coloration, and enriched nutrients. In contrast, the American lotus is predominantly wild-growing, exhibiting a shorter stature and yellow single-petaled flowers [2]. Despite their geographical separation across the Pacific Ocean and distinct phenotypic variations, the two species do not exhibit reproductive isolation, with both possessing eight pairs of diploid chromosomes (2n = 16) [2].

Due to its diverse agronomic characteristics and cultivation purposes, the Asian lotus has been classified into three types over its long history of domestication: seed-lotus, flower-lotus, and rhizome-lotus [3]. Among these, the seed-lotus has attracted particular attention due to its emphasis on seed production. The seed of the lotus, commonly known as “lotus seeds”, holds significant nutritional and medicinal value. These seeds not only serve as a vital reproductive carrier for the lotus but also play a crucial role in human health and cultural heritage. As a key medicinal ingredient in traditional Chinese medicine, lotus seeds are widely recognized for their calming and nourishing properties, among other therapeutic applications [4]. Additionally, their unique taste and rich nutritional components—such as starch, proteins, vitamins, and essential minerals—make them a highly cherished ingredient in various culinary traditions. Fresh lotus seeds exhibit a delicate sweetness and are commonly consumed directly as fruits or used in desserts, whereas dried lotus seeds have been widely applied in the food, pharmaceutical, and health supplement industries, often incorporated into soups, congees, herbal formulations, and nutraceutical products [2,5].

Over the past few decades, the annotation and assembly of the lotus genome have led to significant advancements in molecular biology research on lotus seeds. For example, several genes associated with the edible and medicinal properties of lotus seeds have been identified and functionally validated. Additionally, the molecular mechanisms underlying postharvest preservation and seed longevity have been preliminarily elucidated. These discoveries have not only deepened our understanding of lotus seeds but also attracted increasing attention from researchers to these unique plant seeds. Further in-depth studies into the molecular biology of lotus seeds will aid in elucidating the molecular mechanisms behind seed development, nutrient accumulation, and the formation of their superior qualities. Such research is also crucial for advancing the genetic improvement of lotus seed varieties and fostering the development and utilization of lotus seed resources in sustainable agriculture and functional food industries. This review aims to systematically summarize the latest advancements in the molecular biology of lotus seeds and provide a reference for future fundamental research and industrial application-oriented studies.

## 2. The Typical Structure of Lotus and Its Application Value

**Figure 1:**
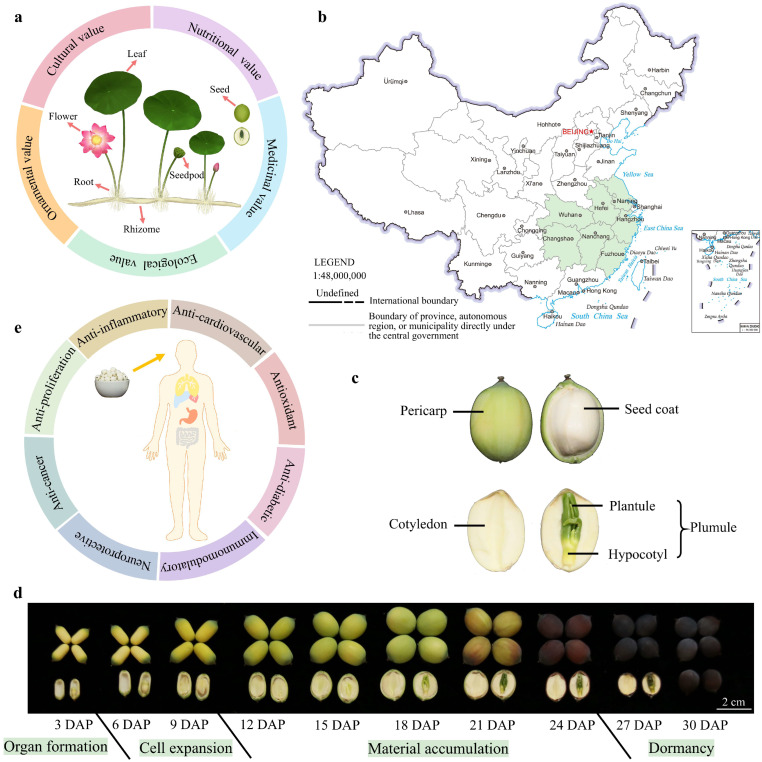
Values and structural characteristics of lotus seeds. (**a**) Typical structural characteristics and application value of lotus, depicting the leaf, flower, seedpod, seed, root, and rhizome. (**b**) Major cultivation area of seed-lotus in China, including Hubei, Hunan, Jiangsu, Jiangxi, Anhui, and Zhejiang provinces. This figure references the standard map with censor code: GS(2019)1673. The diagram is adapted from Sun et al. 2025 [2]. (**c**) Anatomical structure of lotus seeds. (**d**) Morphological changes in lotus seed during development (variety JX, ‘Jianxuan17’). Scale bar = 2 cm. (**e**) Schematic illustrating the benefits of lotus seed consumption.

Lotus is a plant whose entire plant is utilizable. Nearly every part possesses significant application value, including flowers, leaves, seedpods, seeds, rhizomes, and roots (Figure 1a) [2]. The lotus leaf is peltate-shaped, with a thick waxy layer and lipid papillae forming a micro- and nanohierarchical composite structure on its surface. This unique structure endows the leaf with superhydrophobicity and low adhesive properties, preventing water penetration and enabling the self-cleaning phenomenon known as the ‘lotus effect’ [6,7]. The lotus flower is solitary and bisexual, consisting of calyx, stamen, pistil, receptacle, and petal. The petals vary significantly in size, color, shape, and number, resulting in a diverse array of floral morphologies that contribute to the aesthetic and reproductive characteristics of lotus. The seedpod, which is the receptacle of the flower, is conical and features numerous scattered, honeycomb-like pores on its surface. Lotus seeds develop within these pores, each containing a single seed. The rhizome serves as a reservoir for nutrient storage and as a medium for vegetative propagation. When fractured, it reveals white, highly elastic filaments, demonstrating its structural resilience and functional adaptability. The roots emerge from the rhizome nodes, anchoring the plant and facilitating the uptake of water and nutrients.

The lotus is a representative plant with both medicinal and edible properties. Its seeds and rhizomes are edible and can be used in a variety of delicious dishes. Additionally, all tissues of the lotus are utilized in traditional medicine [2]. For example, the lotus plumule is rich in alkaloids like liensinine and isoliensinine, which are employed in treating nervous disorders and arrhythmia [2,8]. Lotus leaves offer a range of therapeutic functions, including heat-clearing, hemostasis, weight reduction, and lipid-lowering effects [9,10]. Furthermore, lotus pollen is known for its antioxidant properties and its skin-beautifying effects. Beyond its ornamental, nutritional, and medicinal values, the lotus also holds significant cultural and ecological importance [2] (Figure 1a). In India, the lotus is regarded as a symbol of sanctity, purity, and beauty [11]. Similarly, ancient Chinese texts frequently highlight the lotus, emphasizing its cultural symbolism. Ecologically, the lotus plays a crucial role in water purification and is widely used in landscape gardening. Its presence in aquatic habitats demonstrates extensive advantages in the ecological restoration of water bodies, making it a valuable component in the management and enhancement of freshwater ecosystems.

### 2.1. Structure and Properties of Lotus Seeds for Edible and Medicinal Purposes

The seed-lotus is an important economic crop in China, with a cultivation area of 2 million mu and an annual output reaching 200,000 metric tonnes. The major cultivation areas are concentrated in provinces such as Jiangsu, Jiangxi, Hunan, Hubei, Zhejiang, and Fujian [12] (Figure 1b). The lotus seed pericarp is formed through the direct thickening of the ovary wall, while the seed coat, cotyledon, and plumule—collectively referred to as the “embryo”—develop from the zygote (Figure 1c). The complete development cycle of lotus seeds lasts approximately 30 days and can be divided into four stages: organ formation (1–3 days after pollination, 1–3 DAP), cell expansion (4–9 DAP), material accumulation (10–25 DAP), and maturation (26–30 DAP) [13]. As lotus seeds develop, the pericarp transitions from green to dark brown and gradually becomes firmer, providing mechanical protection. After dehydration and maturation, the seed coat adheres tightly to the cotyledons, which accumulate substantial starch, with the green plumule enclosed within (Figure 1d). Wang et al. [14] employed metabolomics and proteomics to investigate the metabolic changes occurring during lotus seed development, revealing a significant metabolic transition at 15 DAP from a phase of high metabolic activity to one of material storage. Li et al. [15] conducted a comparative transcriptomic analysis of the development stages of the ‘China Antique’ and ‘Jianxuan 17’ lotus varieties, explored the potential regulatory network between seed size and number, and identified candidate genes. This research provides a crucial foundation for future studies on lotus seed yield and the breeding of high-yield lotus varieties. In the lotus seed cotyledons, transient overexpression of the *NnEXPA2* gene has been shown to promote cell expansion during the early stages of cotyledon development [16]. Additionally, the *NnMSD1* gene is highly expressed in the lotus plumules, responding strongly to low temperature and oxidative stress [17]. Despite these insights, research on the molecular mechanisms underlying lotus seed development remains limited. Unraveling these mechanisms is crucial for identifying high-yielding lotus cultivars and holds significant implications for future breeding programs.

In Asian countries, lotus seeds are predominantly marketed in their dried form and are commonly used in the preparation of congee, pastries, and other traditional dishes. Fresh lotus seeds have a short shelf life and are consumed as a seasonal snack during the summer months [18]. They are packed with a wide range of bioactive compounds and essential nutrients, containing abundant starch, protein, vitamins, and essential amino acids that are vital for the human body, as well as key minerals such as calcium, phosphorus, iron, and zinc. Additionally, lotus seeds are a significant source of bioactive components, including alkaloids, soluble polysaccharides, flavonoids, and phenolic compounds [2]. These compounds collectively provide various health benefits, such as antioxidant, anti-inflammatory, immunomodulatory, and anti-proliferation effects [19] (Figure 1e). As a result, lotus seeds are widely used in both culinary and medicinal applications, making them highly favored by consumers. Here are the latest molecular advancements in the biosynthetic pathways of key bioactive compounds found in lotus seeds, with a particular focus on starch, flavonoids, and benzylisoquinoline alkaloids.

### 2.2. The Starch Biosynthesis and Sugar Metabolism in Lotus Seed

**Figure 2:**
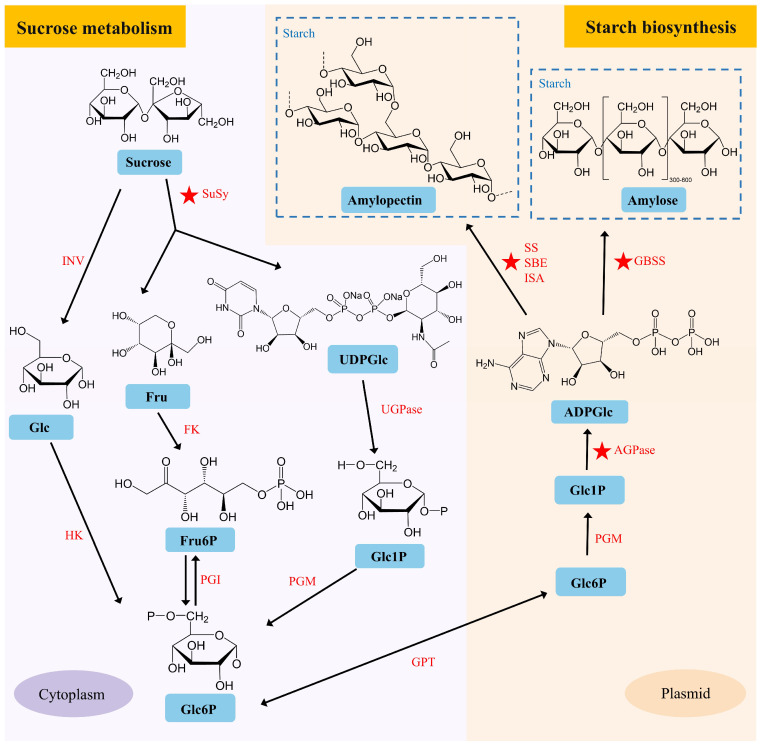
Predicted pathways of starch and sucrose metabolism in lotus cotyledons. Glc, Glucose; Fru, Fructose; UDPGlc, UDP-glucose; Fru6P, Fructose 6-phosphate; Glc1P, Glucose 1-phosphate; Glc6P, Glucose 6-phosphate; ADPGlc, ADP-glucose; INV, Invertase; SuSy, Sucrose Synthase; HK, Hexokinase; FK, Fructose Kinase; PGI, Plastidial Phosphoglucose Isomerase; PGM, Plastidial Phosphoglucomutase; GPT, Glucose-Phosphate Transporter; AGPase, ADP-Glucose Pyrophosphorylase; GBSS, Granule-Bound Starch Synthase; SS, Soluble Starch Synthase; SBE, Starch Branching Enzyme; ISA, Isoamylase. The ★ symbol indicates reported functional genes.

Lotus seed starch (LSS) is a natural high-amylose starch that exhibits unique physicochemical properties, such as a high gelatinization temperature and rapid retrogradation. It has potential applications in the development of foods with low glycemic indexes, prebiotics, and functional films, with broad uses in food processing, healthcare, and the materials industry [20,21]. The cotyledons of lotus seeds primarily accumulate starch, which accounts for approximately 40–60% of the dry biomass. The starch biosynthesis process is crucial during lotus seed development. Starch biosynthesis in developing seeds usually begins with sucrose metabolism [22]. Sucrose is converted into glucose-related substrates via two distinct metabolic pathways, yielding the key intermediate glucose-6-phosphate (Glc6P). Glc6P is subsequently transported into plastids and further converted to ADP-glucose (ADPGlc), which serves as the direct donor substrate for starch polymerization (Figure 2).

The fine structure of starch, including amylose and amylopectin, is determined by a series of catalytic enzymes in the starch biosynthesis pathway, such as granule-bound starch synthase (GBSS), starch-branching enzyme (SBE), and starch synthase (SS) [23]. Starch accumulates rapidly in the cotyledons from 9 to 20 days after pollination [15]. Proteomic and metabolomic profiles show significant metabolic shifts at 15 DAP, transitioning from highly active metabolism to material storage [24]. Comparative transcriptomic analysis revealed that genes encoding enzymes related to starch biosynthesis, such as ADP-glucose pyrophosphorylase (AGPase), SBE, and SS, were upregulated during lotus seed development [15]. Sun et al. [13] described the variation in starch content of lotus seeds among 30 lotus varieties and identified key candidate genes, *NnAGPL2a* and *NnAGPS1a*, that regulate starch biosynthesis. SBE is the sole enzyme capable of introducing α-1,6-glucosidic linkages into α-polyglucans and plays a crucial role in determining the branching density during starch biosynthesis. Zhu et al. [25] cloned two *SBE*-encoding genes, *NnSBEI* and *NnSBEIII*, from lotus seeds. Notably, *NnSBEI* exhibited higher binding activity and a wider affinity for both amylopectin and amylose. Song et al. [20] demonstrated that transient overexpression of the *NnSBE1* gene in lotus seed cotyledons significantly increased starch content. Additionally, various variation sites within the *NnSBE1* gene have led to the formation of distinct haplotypes in seed-lotus compared to other lotus varieties. There have been few reports on the transcriptional regulation of starch synthesis in lotus seeds. Recently, a transcriptional regulatory cascade involving NnLOL1-*NnSBE1* has been reported to participate in starch synthesis. *NnLOL1* encodes a C2H2-type zinc finger protein, which positively regulates the expression of the *NnSBE1* gene and is involved in starch synthesis in lotus seeds [20].

Progress has also been made in the study of genes involved in sugar metabolism in lotus seeds. In non-photosynthetic tissues, sucrose is converted into fructose and UDP-glucose by the enzyme sucrose synthase (SuSy). Transient overexpression of the SuSy encoding gene *NnSUS1* in lotus seed cotyledons significantly increased the total soluble sugar content (including sucrose and fructose), highlighting its key role in sugar accumulation in lotus seeds [26]. The Sugars Will Eventually be Exported Transporter (SWEET) proteins are a family of sugar transporters responsible for facilitating the movement of sugars across cell membranes. Transient overexpression of the SWEET gene *NnSWEET14* in lotus seed cotyledons significantly increased soluble sugar content [3]. Accelerating the functional characterization of genes related to lotus seed starch biosynthesis and sugar metabolism could facilitate the application of molecular breeding techniques in developing new lotus varieties with enhanced starch and sugar contents in the future.

### 2.3. Biosynthesis of Benzylisoquinoline Alkaloids in Lotus Seed

Alkaloids are a class of secondary metabolites with a broad range of pharmacological activities, including antioxidant, anti-inflammatory, anti-proliferative, anti-cancer, and anti-cardiovascular effects. These properties make them highly promising candidates for developing novel therapeutic agents targeting various life-threatening diseases, such as microbial infections, inflammation, atherosclerosis, cancer, obesity, neurological disorders, and diabetes [2]. The alkaloids found in lotus are primarily distributed in the leaves and plumules and all belong to the benzylisoquinoline alkaloids (BIAs) group, which can be classified into monobenzylisoquinoline, bis-benzylisoquinoline alkaloids (bis-BIAs), and aporphine alkaloids [2,8]. Functional group modifications, particularly *O*- and *N*-methylation, as well as C-C and C-O coupling reactions, drive the diversification of BIAs, resulting in additional structural subclasses [27,28]. The lotus plumules mainly accumulate bis-BIAs, such as liensinine, neferine, and isoliensinine, while the lotus leaves mainly contain aporphine alkaloids, including nuciferine, *O*-nornuciferine, and *N*-nornuciferin [2]. Notably, the BIAs accumulated in lotus exhibit a prevalence of *R*-enantiomers, including proaporphine, aporphine, and bisbenzylisoquinoline structural subgroups. This contrasts with the dominant *S* configuration observed in the Ranunculaceae family [29,30].

**Figure 3:**
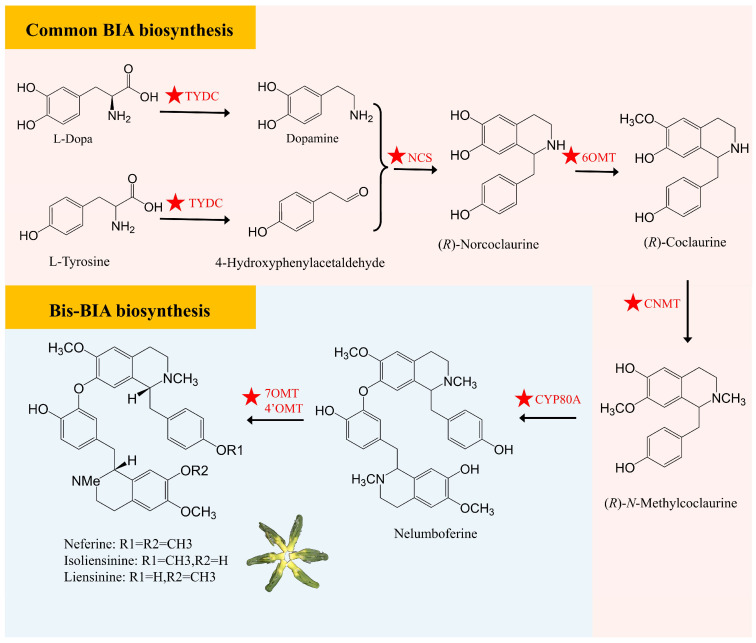
Predicted biosynthetic pathway of bis-BIAs in lotus plumule. TYDC, Tyrosine Decarboxylase; NCS, Norcoclaurine Synthase; 6OMT, Norcoclaurine 6-*O*-Methyltransferase; CNMT, Coclaurine *N*-Methyltransferase; CYP80A, (*S*)-*N*-methylcoclaurine 3′-hydroxylase; 7OMT, 7-*O*-Methyltransferase; 4′OMT, 4′-*O*-Methyltransferase. The ★ symbol indicates reported functional genes.

The synthesis of bis-BIAs in lotus seed plumules begins with two molecules of L-tyrosine metabolism and involves multiple key enzyme genes. Several alkaloid synthesis-related genes, such as *TYDC*, *NCS*, *OMT*, and *CYP80A*, have been identified, and they play crucial roles in the biosynthesis of lotus plumule alkaloids. Ultimately, three main bis-BIAs, including liensinine, isoliensinine, and neferine, are produced through the catalysis of 4′OMT or 7OMT enzymes [29]. Other enzyme modifications, including *N*-demethylation, double 4′-*O*-methylation, and 8-*O*-3′ and 3–3′ intermolecular coupling, contribute to the formation of various bis-BIA structures [2] (Figure 3). Several studies have elucidated the catalytic activities of NnCYP80A and NnCYP80G, which are pivotal enzymes in the biosynthesis of bis-BIAs and aporphine alkaloids in lotus, respectively. Specifically, NnCYP80A catalyzes the conversion of (*R*)-*N*-methylcoclaurine to nelumboferine, while NnCYP80G converts the same substrate to glaziovine [29,30]. *NnCYP80A* is highly expressed in lotus plumules and mediates C-O coupling reactions in both (*R*)- and (*S*)-*N*-methylcoclaurine, leading to the formation of bis-BIAs with diverse linkages. In contrast, *NnCYP80G* is expressed primarily in the lotus laminae and is involved in aporphine alkaloid biosynthesis, catalyzing C-C coupling reactions and demonstrating notable substrate versatility through the utilization of (*R*)-*N*-ethylcoclaurine [8,31]. Zhang et al. [8] demonstrated that NnMYC2, a core regulator in the jasmonic acid (JA) signaling pathway, functions upstream of NnMYB14. The NnMYC2-NnMYB14 transcription factor module specifically and directly binds to the promoters of the *NnCYP80G* and *NnCYP80A* genes, thereby positively regulating their transcriptional expression and the lotus BIA biosynthesis. Additionally, Sun et al. [32] employed RNA-seq technology to reveal the dynamic expression changes in structural genes related to bis-BIA biosynthesis in lotus plumules, which found differentiation and redundancy in the functions of some structural genes. Through functional analysis of these key enzyme genes and their expression regulation mechanisms, it is expected that the alkaloid biosynthesis pathway in lotus plumules will be further optimized.

### 2.4. Biosynthesis of Flavonoids in Lotus Seed

Flavonoids are a group of plant polyphenols known for their rich chemical diversity and a wide range of pharmacological effects, including antioxidant, anti-inflammatory, anti-obesity, and hemostatic activities [33,34]. Several techniques have been established to determine the content and composition of flavonoids in different lotus tissues, including high-performance liquid chromatography with diode array detector (HPLC-DAD), ultra-performance liquid chromatography with tandem mass spectrometry (UPLC-MS/MS), and ultra-performance liquid chromatography-quadrupole time-of-flight mass spectrometry (UPLC-ESI-QTOF-MS) [35,36,37,38]. Flavonoids in lotus are primarily categorized into two major categories: *O*-glycosides, which accumulate mainly as flavones and flavonols, and *C*-glycosides, which accumulate as flavonoid *C*-glycosides. Both types can be found in the plumules of lotus seeds [38,39,40,41]. It is noteworthy that the plumules of lotus seeds predominantly accumulate flavonoid *C*-glycosides, while the leaves are exclusively composed of flavonoid *O*-glycosides [42]. Flavonoid *C*-glycosides are characterized by the direct linkage of sugars to the flavonoid backbone through C-C bonds, making them more stable than flavonoid *O*-glycosides, which are linked by C-O bonds. These *C*-glycosides possess significant medicinal and health benefits, including pharmacological activities such as antioxidant, anti-inflammatory, anti-tumor, anti-diabetic, and antiviral effects [43]. Zhu et al. [42] used HPLC-MS to identify eight flavonoid *C*-glycosides and eight *O*-glycosides from lotus plumule. Among them, luteolin 7-*O*-neohesperidoside and kaempferol 7-*O*-glucoside were identified for the first time. Furthermore, 16 flavonoids, including nine flavonoid *C*-glycosides (e.g., orientin and vitexin) and seven flavonoid *O*-glycosides (e.g., rutin and quercitrin), were detected in the plumule of four representative lotus cultivars using a combination of macroporous adsorption resin chromatography with liquid chromatography with ultraviolet detection/liquid chromatography-mass spectrometry (LC-UV/LC-MS) [39]. Currently, research on lotus flavonoids primarily focuses on purification techniques and pharmacological activities, with limited exploration of their biosynthesis. This has resulted in slow progress in breeding high-flavonoid-content varieties.

**Figure 4:**
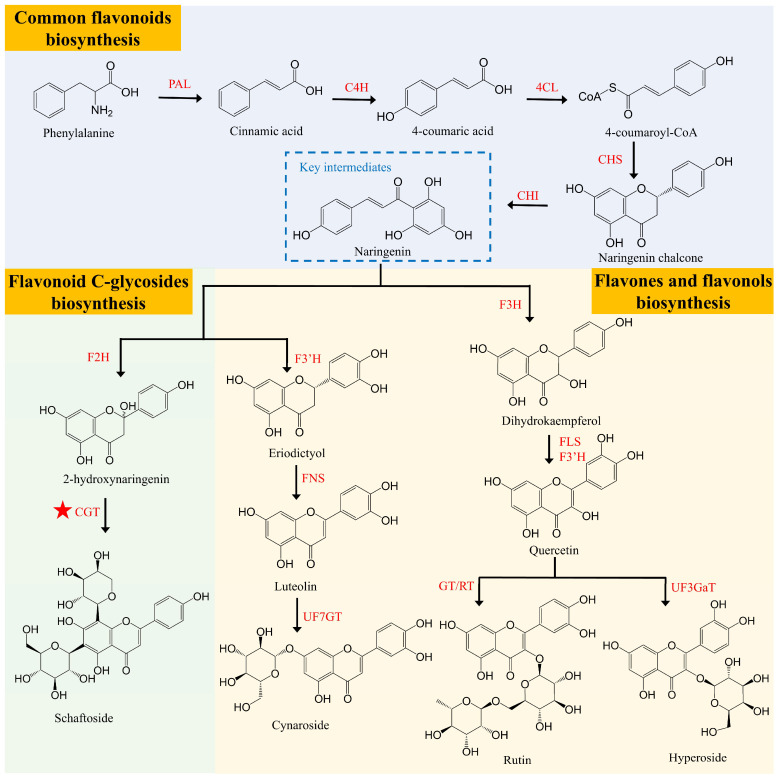
Predicted biosynthetic pathway of flavonoid in lotus plumule. PAL, Phenylalanine Ammonia Lyase; C4H, Cinnamate 4-Hydroxylase; 4CL, 4-Coumaryol CoA Ligase; CHS, Chalcone Synthase; CHI, Chalcone Isomerise; F3H, Flavanone 3-Hydroxylase; FLS, Flavonol Synthase; F3′H, Flavonoid 3′-Hydroxylase; UF3GaT, UDP-galactose: flavonoid 3-*O*-galactosyltransferase; GT, Glucosyltransferase; RT, Rhamnosyltransferase; F2H, Flavanone 2-Hydroxylase; CGT, Cyclodextrin Glycosyltransferase; FNS, Flavone Synthase; UF7GT, UDP-glucose: flavanone 7-*O*-glucosyltransferase. The ★ symbol indicates reported functional genes.

The biosynthesis of flavonoids in lotus is both complex and conserved. It initiates from phenylalanine, which, through the action of enzymes such as PAL, C4H, 4CL, CHS, and CHI, is converted into naringenin. Naringenin acts as a crucial intermediate carrier in the formation of flavonoids. Subsequently, under the catalysis of enzymes like F3H, F2H, and CGT, various flavonoid compounds with distinct types and biological activities are produced [33] (Figure 4). Feng et al. [44] identified two glycosyltransferases, UGT708N1 and UGT708N2, involved in the synthesis of flavonoid *C*-glycosides in lotus plumules. UGT708N1 catalyzes the conversion of 2-hydroxy naringenin and 2-hydroxy eriodictyol into corresponding monocarbon glucosides, which are then converted into flavonoid monocarbon glycosides through a dehydration reaction. UGT708N2 further catalyzes the arabinosylation or xylosylation of 2-hydroxy naringenin monocarbon glucoside, followed by a dehydration reaction that generates flavonoid dicarbon glycosides. Song et al. (unpublished data) demonstrated that transient overexpression of *NnCGTa* in lotus petals significantly enhanced the accumulation of flavonoid *C*-glycosides. Despite the conservation and complexity of flavonoid biosynthesis in lotus, research on its transcriptional regulatory mechanisms remains limited. A recent study has characterized the R2R3-MYB transcription factor, NnMYB12, which exhibits high expression in lotus plumules. Its transient overexpression markedly elevated flavonoid levels and the expression of biosynthetic genes in both lotus petals and tobacco leaves. NnMYB12 was shown to directly bind to the *NnF3′H* promoter, activating its transcription and thereby mediating flavonoid biosynthesis in lotus plumules (Song et al., unpublished data). These findings provide valuable insights for the synthetic biological production of lotus flavonoid *C*-glycosides.

### 2.5. Molecular Mechanisms of Lotus Seed Postharvest Preservation

The harvest of lotus seeds primarily occurs during the summer months, specifically from July to September [45]. Under high-temperature storage conditions, the fresh lotus seeds after harvest undergo vigorous metabolism, which causes their pericarp to brown quickly and accelerates the quality deterioration. Tu et al. [46] compared the physicochemical properties and sensory qualities of two fresh lotus seed varieties, ‘Mantianxing’ and ‘No.36 space lotus’, and found that the harvest time of fresh lotus seeds directly affects their taste and flavor. Fresh lotus seeds, often used as a fruit snack, are typically harvested around 15 DAP, when soluble sugars are predominantly accumulated, imparting a sweet taste. However, as seeds mature, rapid starch synthesis in the cotyledon and bis-BIAs in the plumule results in a firmer texture and bitterness [13,47]. Yang et al. [47] further discovered that the decrease in fructose content is attributed to the upregulation of fructokinase (FRK) activity and related gene expression. Additionally, the reduction in malic acid content is associated with the diminished activity of phosphoenolpyruvate carboxylase (PEPC) and NAD-malate dehydrogenase (NAD-MDH). Polyphenol oxidase (PPO) is the primary cause of enzymatic browning, which leads to changes in color, texture, taste, and nutritional components, resulting in significant losses in food quality and nutritional value [48]. Fresh lotus seeds contain high polyphenol levels, and the browning caused by internal PPO deteriorates their quality, taste, and flavor, leading to nutrient losses exceeding 50% [49]. Chen et al. [50] identified an up-regulated gene co-expression module associated with pericarp phenotypic changes via weighted gene co-expression network analysis (WGCNA) and characterized two PPO-encoding genes (*NnPPO2* and *NnPPO3*), whose expression levels and enzymatic activities are closely correlated with the deterioration of lotus seed appearance quality.

Ongoing research has focused on understanding the internal mechanisms behind the rapid decline in postharvest quality of fresh lotus seeds and exploring preservation techniques. Postharvest physiological and transcriptomic analyses of ‘Jianxuan 17’ lotus seeds identified 3148 differentially expressed genes (DEGs), which were primarily enriched in the starch and sucrose metabolism pathway. Moreover, the rapid increase in starch and protein content, coupled with the continuous decrease in soluble sugar content during storage, are key factors contributing to the decline in lotus seed quality [5]. RNA sequencing of postharvest lotus seeds stored at room temperature versus low temperature revealed fewer DEGs in the latter, with significant suppression of ABA signaling-related gene expression, indicating that low temperature effectively inhibits the deterioration process [51].

Research has also demonstrated that the use of preservatives is an effective strategy to slow the decline in lotus seed quality. For example, the ethylene inhibitor 1-Methylcyclopropene (1-MCP) can effectively delay postharvest physiological deterioration and maintain the storage quality of many fruits and vegetables by inhibiting respiration, improving texture, and reducing microbial growth [52,53]. Treating fresh lotus seeds with 1-MCP demonstrated reduced browning of fresh lotus pods and pericarps and inhibited the respiration rate during storage [54]. In another study, treating lotus seeds with 0.5 μL/L 1-MCP and 3% lacquer wax revealed that each treatment alone could reduce pericarp browning and maintain short-term freshness. The combined treatment effectively delayed cell wall degradation and plasmolysis, preserving cell integrity [55]. Chen et al. [56] evaluated the inhibitory effects of three preservatives, ascorbic acid (AA), benzoic acid (BA), and sodium sulfite (SDS), and their combinations on lotus seed browning. They found that a combination of 0.25 g/100 mL SDS and 0.25 g/100 mL AA effectively inhibited browning. Luo et al. [45] treated fresh lotus seeds with 6-benzylaminopurine (6-BA) and melatonin (MT), respectively, and stored them at 25 ± 1 °C for 8 days. The results showed that 6-BA treatment might influence the activities of enzymes involved in starch and sucrose metabolism, thereby maintaining starch and sucrose levels and slowing postharvest deterioration. In contrast, MT treatment increased endogenous MT levels, decreased cell membrane permeability, ensured energy supply and membrane integrity, inhibited respiration and browning, and mitigated postharvest senescence in lotus seeds [18]. Further research indicated that combining nitric oxide (NO) and MT effectively inhibited mitochondrial ROS generation, increased respiratory enzyme activities, enhanced ATP production, and delayed browning [57]. Additionally, Xu et al. [58] developed an antibacterial package that extended the shelf life of fresh lotus seeds to 25 days when stored at 4 °C, effectively delaying postharvest senescence. Chi et al. [59] developed a rapid freshness detection method using desorption atmospheric pressure chemical ionization-mass spectrometry (DAPCI-MS), enabling accurate identification of lotus seed freshness. Future research into the preservation mechanisms of lotus seeds not only addresses the regional and seasonal limitations of lotus seed availability but also provides substantial support for their processing and marketing, holding significant market application potential.

### 2.6. Molecular Mechanisms of Lotus Seed Longevity

Seeds play a crucial role in the plant life cycle, and their longevity is a key factor determining their long-term storage [60,61]. Lotus seeds are among the longest-lived seeds in the world, possessing exceptional vitality. They can survive for thousands of years in the peat layers of the natural environment and germinate under suitable conditions [62]. The several century-old ancient lotus seeds have been successfully germinated under the circumstance of one end of the seeds being filed down to facilitate water penetration and imbibition [63]. Sun et al. [64] employed the identical germination method and achieved successful germination of ancient lotus seeds dated back to 610 ± 25 years before the present (BP). The longevity of lotus seeds is closely related to their unique structure and physiological mechanisms. The pericarp of lotus seeds has a dense structure and is covered by a waxy layer, forming a critical barrier between the seeds and the external environment [64,65]. Lotus seeds also exhibit notable thermotolerance. During the initial exposure to 100 °C, the activities of superoxide dismutase (SOD) and catalase (CAT) increase before declining [66]. The CuZn-SOD enzyme effectively scavenges ROS under extreme temperatures, thereby preserving the physiological activity of lotus seeds and maintaining the integrity of cellular structures [65]. Sun et al. [64] further confirmed the heat resistance of lotus seeds. Even after being treated at 75 °C for 12 h, intact RNA nucleotides can still be extracted, and the germination rate remains at 60%. Lotus seeds have evolved exceptional heat tolerance, presumably as an adaptive response to their natural habitat and reproductive strategy, which synergizes with their thick lignified pericarp to protect embryonic cells and maintain seed viability during long-term dormancy and environmental fluctuations. Furthermore, the longevity mechanism of lotus seeds involves the synergistic action of various heat-stable proteins. For example, the ectopic expression of the heat-induced annexin encoded by the *NnANN1* gene in transgenic *Arabidopsis* significantly enhances seed thermotolerance and peroxidase activity [67]. Proteins in the cotyledons and plumules, such as heat shock protein 80 (HSP80), protein l-isoleucine methyltransferase 1 (PIMT1), and methionine synthase, maintain fluidity even at a temperature as high as 110 °C, which may contribute to the long-term survival of the lotus seeds [65]. Additionally, the NnCAT protein alleviates oxidative stress damage to cells by scavenging ROS [68].

**Figure 5:**
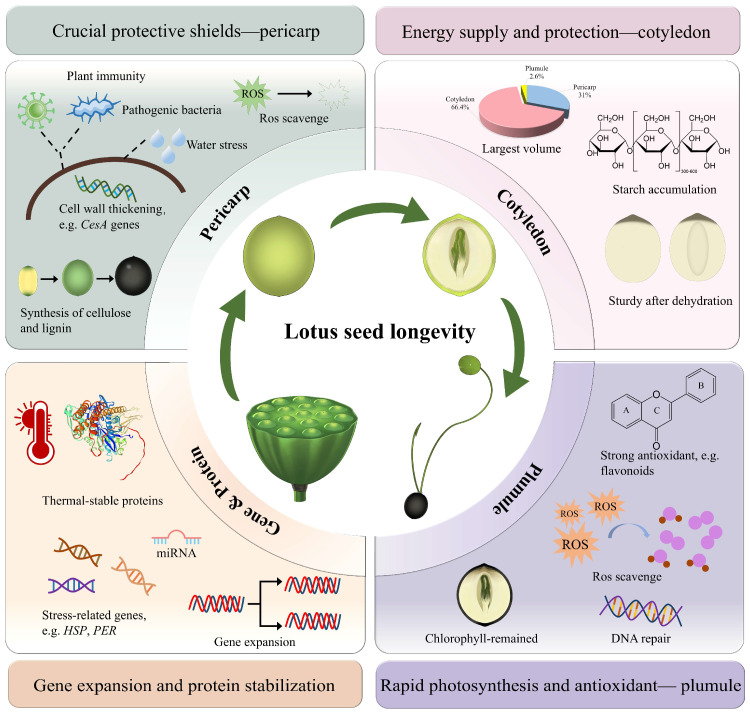
A schematic illustration elucidating the multifaceted mechanisms underlying the lotus seed longevity. The diagram is drawn based on Sun et al. 2025 [64].

Recent years have seen substantial progress in understanding the longevity mechanisms of lotus seeds through gene function and molecular biology studies. Heterologous expression of the cytoplasmic class II small heat shock protein gene *NnHSP17.5*, along with the metallothionein genes *NnMT2a* and *NnMT3* in *Arabidopsis*, has been shown to significantly improve seed germination viability, with *NnHSP17.5* also conferring thermotolerance to seedlings [69,70]. Lotus contains eight antioxidant 1-cys peroxidase (PER) genes. Overexpressing *NnPER1* in *Arabidopsis* reduces DNA damage and lipid peroxidation, thereby improving seed stability and extending seed longevity [64,71]. Chen et al. [1] conducted a genome-wide identification of the *NnLEA* gene family in lotus and found that most *NnLEAs* are highly expressed in the late stages of lotus seed development, indicating their crucial role in the dehydration stress response. Additionally, *HSP* genes related to cell homeostasis maintenance and protein folding may protect against dehydration-induced stress during lotus seed maturation [24,64]. A recent study explored the internal mechanisms behind the longevity of lotus seeds from physiological, biochemical, and molecular perspectives. The results indicated that the tissue structure of lotus seeds plays an essential role in their longevity [64]. The robust structure of the pericarp, along with its high content of antioxidants such as flavonoids and polyphenols, provides protection and high antioxidant activity. The starch-rich cotyledons and chlorophyll-retaining plumules not only provide sufficient energy for long-term storage and germination but also facilitate rapid photosynthesis post-germination [64]. The survival of lotus seeds over centuries offers an exemplary model for studying seed adaptation mechanisms [72]. Sun et al. [64] found that the number of genes related to seed maturation and defense response in the lotus genome significantly expands under prolonged adverse storage conditions, which may be the result of adaptive evolution. Furthermore, results from high-throughput miRNA sequencing and degradome analysis indicate that the germination of lotus seeds is regulated by miRNAs, providing novel insights for future research on longevity mechanisms [73]. In conclusion, the remarkable longevity of the lotus seed is facilitated by the contributions of its intrinsic structural components, as well as the complex regulation of genes and protein functions (Figure 5).

## 3. Opportunities and Perspectives

Lotus seeds, the unique dual-function propagules (possessing both fruit and seed attributes) of *Nelumbo* plants, have evolved over millennia of domestication and cultivation into a distinctive agricultural heritage in Asia [4]. Due to their high starch content and rich flavor profile, lotus seeds have become deeply integrated into the East Asian diet, serving as a typical example of “food-medicine homology”. Lotus plumules are rich in bioactive compounds (e.g., flavonoids, alkaloids), with extensive biological activities and pharmacological functions (Table S1) [2,35,38,74,75,76,77,78,79,80,81], which play significant roles in traditional medicine, biopharmaceuticals, and disease treatment [2]. However, due to the regional distribution of *Nelumbo* plants, this valuable resource remains largely underappreciated.

**Figure 6:**
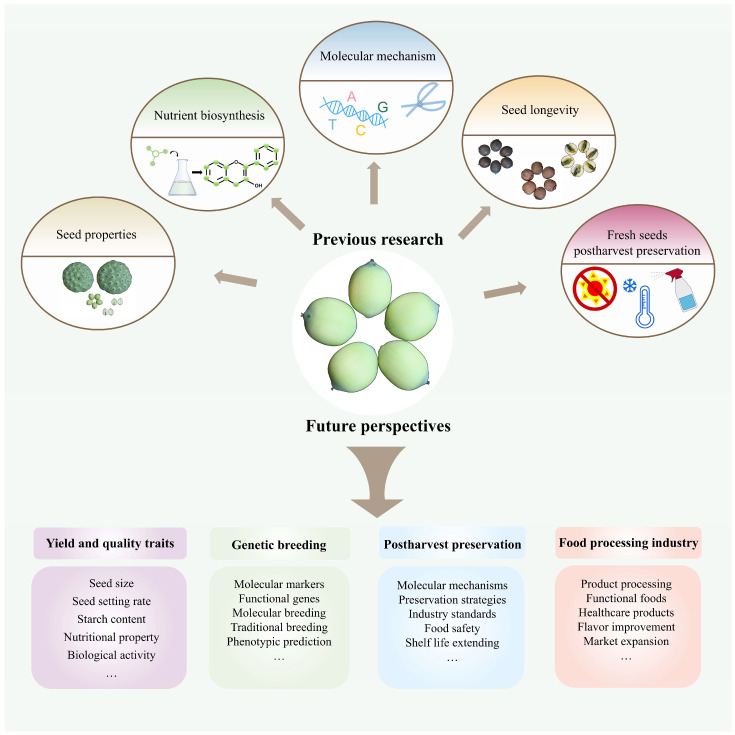
A summary model for previous research and future perspectives on lotus seed.

This review elucidates the molecular mechanisms underlying the biosynthesis of key nutrients, including starch, alkaloids, and flavonoids, and highlights the identification and functional studies of related genes that contribute to the functional and medicinal properties of lotus seeds (Table S2). Additionally, it systematically summarizes molecular studies on the postharvest preservation of fresh lotus seeds and the mechanisms underlying lotus seed longevity (Figure 6). Currently, molecular research on lotus seeds is still in a nascent stage. Future work should focus on unraveling the biological characteristics of lotus seeds, exploring their potential value, clarifying the biosynthetic pathways of key nutrients, and advancing innovations in postharvest preservation technologies for fresh lotus seeds. Furthermore, integrating modern molecular techniques is essential to accelerate the development of new lotus varieties, thereby facilitating the sustainable growth of the lotus seed industry.

### 3.1. Development of Application Potential of Lotus Seeds and Breeding Strategies

The lotus seeds, renowned for their high amylose starch content, have significant applications across the food, pharmaceutical, and industrial sectors. As a low-Glycemic Index (low-GI) food, lotus seed starch benefits gut health by regulating the gut microbiota, making it particularly beneficial for diabetic patients [82]. Moreover, lotus seed resistant starch promotes the proliferation of beneficial probiotics (e.g., *Lactobacillus* and *Bifidobacterium*) while inhibiting harmful bacteria [83]. Enhancing starch synthesis in lotus seeds holds potential for yield improvement and significant economic gains. Mature lotus seeds are also rich in proteins and polysaccharides, providing exceptional nutritional value. Notably, they exhibit remarkable longevity, with some remaining viable for centuries under optimal storage conditions [62,64]. This resilience positions lotus seeds as a crucial resource for food security. Elucidating the molecular mechanisms underlying this longevity could offer strategies to enhance seed durability and resilience, ultimately supporting sustainable food systems and global food security.

A key area of research in lotus seed development is the biosynthesis of bioactive compounds such as bis-BIAs and flavonoids in the lotus plumules. Studies have demonstrated that bis-BIA synthesis is mediated by a series of enzymatic reactions, with several key enzyme-encoding genes identified and functionally validated [2,29]. Furthermore, identifying transcription factors regulating bioactive component synthesis and clarifying their regulatory mechanisms will deepen the understanding of alkaloid and flavonoid biosynthesis in lotus plumules. This knowledge lays the groundwork for synthetic biology approaches, enabling the development of artificial biosynthetic routes for these valuable secondary metabolites. Future research may focus on large-scale production using chemical synthesis or biosynthetic methods through microorganisms or plant cell cultures, reducing reliance on natural resources and contributing to environmental conservation. Notably, optimizing the extraction, separation, and identification of bioactive compounds in lotus plumules is crucial for their practical applications. Advanced extraction technologies, such as supercritical fluid extraction (SFE) and high-speed countercurrent chromatography (HSCCC), can significantly improve the efficiency and yield of bioactive compounds [84,85]. Meanwhile, developing new identification methods will ensure precise separation and purification of bis-BIAs and flavonoids, yielding high-purity compounds suitable for therapeutic applications.

Recent advancements in genetics, molecular biology, and bioinformatics have facilitated in-depth exploration of the genetic basis of lotus seed development [72]. Techniques like whole-genome resequencing and genome-wide association studies (GWAS) are essential for identifying key genes that regulate yield and quality traits of lotus seeds, such as seed size, starch and alkaloid contents [86,87]. Additionally, the construction of high-density genetic maps and quantitative trait locus (QTL) mapping has enabled the development of molecular markers, facilitating early identification of desirable seed traits [88,89]. Advancing superior lotus cultivar breeding should integrate traditional breeding techniques with modern biotechnologies, such as hybridization and mutagenesis. Establishing a core germplasm resource bank and strengthening the assessment of genetic diversity will provide abundant genetic materials for the breeding of new varieties. Moreover, developing a rapid and accurate variety identification system will facilitate the timely registration and promotion of new varieties, enhancing the economic and social benefits of lotus seed cultivation.

Lotus is an open-pollinated plant, characterized by protogynous flowers that lack nectar. During the anthesis stage, these fragrant flowers are capable of self-heating, and each flower generates approximately millions of pollen grains. Bees, flies and beetles constitute the primary floral visitors [90,91,92]. This open-pollination mode plays a crucial role in maintaining genetic diversity and seed quality in plants such as maize, wheat, sorghum and eucalyptus. Open pollination has been proven to maintain a high level of heterozygosity and allele richness, which is beneficial for breeding high-quality and widely adapted varieties [93]. It also enhances the seed setting rate, vitality and sizes by mitigating inbreeding depression [94,95]. In lotus, in comparison to the natural pollination, the seed-setting rate of self-pollination was reduced, indicating self-incompatibility [96]. Nevertheless, no research has focused on how open pollination leads to genetic variations among different varieties. Future research can study the effect of open pollination on the development and vitality of lotus seeds, as well as the molecular mechanisms related to the accumulation of nutrients such as starch and flavonoids. This will facilitate a deeper understanding of the biological characteristics and genetic diversity of lotus seeds, providing a theoretical basis for the breeding and quality improvement of lotus seeds. Meanwhile, it also uncovers some unexplored mysteries in the plant reproduction process, which is of great significance for protecting the genetic resources of lotus.

### 3.2. Optimize Postharvest Preservation Techniques of Fresh Lotus Seeds to Extend Shelf Life

The postharvest deterioration of fresh lotus seeds is closely associated with physiological metabolic processes, including respiration, sucrose-starch conversion, and alterations in antioxidant enzyme activity [5,47]. Future research should prioritize the elucidation of the postharvest metabolic network of fresh lotus seeds, especially key pathways such as starch and sucrose metabolism and phenolic compound oxidation. Multi-omics approaches (transcriptomics, proteomics, metabolomics) can comprehensively characterize the dynamics of gene expression, signal transduction, and metabolite profiles during the process of quality deterioration [97,98]. Subsequent functional verification studies of candidate genes will lay a theoretical foundation for targeted genetic regulation. Furthermore, the development of molecular markers associated with postharvest senescence and the establishment of predictive models could facilitate early shelf life, thereby providing theoretical support for optimizing preservation techniques and enabling real-time postharvest quality monitoring.

Exogenous regulators hold substantial potential for postharvest preservation of fresh lotus seeds. Previous studies have demonstrated that 1-MCP, 6-BA, and MT can effectively delay postharvest senescence [52,53,55]. Future research should prioritize the optimization of the concentration, application timing, and delivery methods of these regulators to enhance preservation efficacy, such as through the development of composite preservatives via the synergistic combination of different regulators [55]. Although traditional preservation techniques (e.g., refrigeration, modified atmosphere storage) exhibit effectiveness, they are constrained by practical limitations. Innovative preservation strategies, including bio-preservatives (microbial fermentation or biofilms), intelligent packaging (active packaging and time-temperature indicators), and physical treatments (ultrasound or pulsed electric field treatments), merit further exploration [99,100]. Moreover, the integration of multiple techniques (e.g., low-temperature storage coupled with exogenous regulator application) may further extend the shelf life of fresh lotus seeds. Such research will not only prolong postharvest longevity but also enhance the market value and consumer satisfaction of fresh lotus seeds.

Fresh lotus seeds are highly susceptible to postharvest microbial contamination, which triggers rapid quality degradation and spoilage. Future research should focus on dissecting the dynamic shifts in microbial community structure and their regulatory effects on lotus seed quality. For instance, microbiomic technologies can be employed to analyze the dynamic changes in the postharvest microbial assemblages of lotus seeds [101]. Such analyses can aid in identifying beneficial or antagonistic microorganisms, laying a theoretical foundation for the development of microbial-based preservation strategies. Moreover, investigating the interaction between microorganisms and the endogenous physiological metabolism of lotus seeds will provide novel insights for designing safer and more efficient preservation techniques. Currently, most postharvest preservation technologies for fresh lotus seeds remain confined to laboratory-scale validation. Moving forward, priority should be given to advancing their industrial translation and standardization. Collaborations with industrial stakeholders to develop scalable preservation protocols, coupled with the establishment of unified quality control standards, will be pivotal for promoting the industrialization of fresh lotus seed preservation.

### 3.3. Innovative Processing Technologies for High-Value Lotus Seed Products

Given the abundant nutritional profile and unique medicinal properties of lotus seeds, there is an urgent imperative to intensify research and development efforts focused on health products and innovative food items. Modern food processing technologies, such as ultrafine grinding, nanotechnology, and microencapsulation, can be employed to create high value-added functional lotus seed foods [102,103]. For instance, microencapsulation can protect the flavor and nutrients of lotus seeds, facilitating their incorporation into confectionery products like candies and chocolates. This not only imparts a distinctive lotus seed flavor but also broadens its application in the snack food industry. Incorporating bioactive-rich lotus plumule extracts into baked goods can enhance their nutritional value and create specialized products with health benefits while preserving their unique flavor. Similarly, these extracts can be used as additives in beverages, resulting in functional drinks with invigorating and antioxidant properties, catering to the growing demand for healthy, flavorful food options. Furthermore, lotus plumule extracts can be developed into functional health products (e.g., oral liquids, capsules) for auxiliary management of cardiovascular diseases, lipid regulation, and immune enhancement [104]. Flavonoids found in lotus seeds can help eliminate excess free radicals, boost immunity, and prevent age-related diseases [33]. Incorporating flavonoids into cosmetic formulations can also improve skin antioxidant capacity and reduce UV damage.

The lotus seed processing industry should proactively expand market applications and develop products tailored for diverse consumer groups. Customizable lotus seed products for specific demographics such as pregnant women, the elderly, and fitness enthusiasts can meet the growing demand for natural and safe products. Additionally, processing by-products (e.g., lotus seed pericarps) is rich in bioactive compounds, holding significant potential for value-added utilization, which could boost the overall economic returns of the lotus seed industry. Amid the continuous expansion of the global health food market, the lotus seed processing industry must actively explore international markets. Establishing an integrated production-processing-sales model and strengthening brand development and market promotion, as well as advancing large-scale, industrialized development of the lotus seed processing sector, are critical [105]. Through technological upgrading and market expansion, the industry can elevate the global competitiveness of lotus seeds, maximize economic returns, and solidify the position of lotus seeds as an indispensable component of the global health food supply chain.

## 4. Conclusions

Continuous improvements in lotus genome assembly and annotation have substantially accelerated molecular biology research on lotus seeds. Deciphering the core mechanisms underlying key traits, such as the biosynthesis of nutritional and bioactive compounds (e.g., starch, flavonoids, alkaloids), seed development, postharvest preservation, and exceptional longevity, has identified promising candidate genes for targeted genetic improvement. These advancements not only enhance our comprehension of the distinctive biological characteristics of lotus seeds but also provide robust molecular tools for breeding high-quality varieties with enhanced nutritional value, extended shelf life, and improved agronomic traits. Furthermore, these achievements will also facilitate the sustainable development of the lotus seed industry in the future.

## Data Availability

Data sharing is not applicable to this review, as no datasets were generated or analyzed during the current study.

## Supplementary Materials

The following supporting information can be downloaded at: https://www.mdpi.com/article/10.3390/plants15010136/s1, Table S1 Primary bioactive components of lotus seeds and their functions; Table S2 Genes involved in lotus seed development and their functions; Table S3 Abbreviations of proper nouns.

## References

[1] Chen L., Xin J., Song H.Y., Xu F., Yang H., Sun H., Yang M. (2023). Genome-wide study and functional characterization elucidates the potential association of late embryogenesis abundant (*LEA*) genes with lotus seed development. Int. J. Biol. Macromol..

[2] Sun H., Xin J., Song H.Y., Chen L., Yang D., Yang H., Deng X.B., Liu J., Cui R., Su Y. (2025). Harnessing genomic and molecular biology resources for genetic improvement of lotus: Current achievements and future directions. Hortic. Adv..

[3] Chen L., Xin J., Song H.Y., Cheng W., Yang M., Yang D., Sun H. (2024). Evolution and seed development responses of *Nelumbo SWEET* genes. Sci. Hortic..

[4] Bishayee A., Patel P.A., Sharma P., Thoutireddy S., Das N. (2022). Lotus (*Nelumbo nucifera* Gaertn.) and its bioactive phytocompounds: A tribute to cancer prevention and intervention. Cancers.

[5] Sun H., Liu Y.L., Ma J.Y., Wang Y.M., Song H.Y., Li J.J., Deng X.B., Yang D., Liu J., Zhang M.H. (2021). Transcriptome analysis provides strategies for postharvest lotus seeds preservation. Postharvest Biol. Technol..

[6] Zhang M.Q., Feng S., Wang L., Zheng Y.M. (2016). Lotus effect in wetting and self-cleaning. Biotribology.

[7] Wang L. (2023). A critical review on robust self-cleaning property of lotus leaf. Soft Matter.

[8] Zhang M.H., Xu Z.T., Yang Z.J., Song H.Y., Xin J., Yang H., Sun H., Liu J., Yang D., Liu Y.L. (2025). Spatial regulation of benzylisoquinoline alkaloid biosynthesis in lotus (*Nelumbo nucifera*) is controlled coordinately through the NnMYC2-NnMYB14-NnCYP80 modules. Hortic. Res..

[9] Liu L.P., Han J.L., Tian H.Y., Liu Z., Wang X.Z., Wang P.W., Li X., Gao W.Y. (2025). A review on the progress of research on the chemical composition and pharmacological effects of lotus leaf. Food Chem..

[10] Bharathi Priya L., Huang C.Y., Hu R.M., Balasubramanian B., Baskaran R. (2021). An updated review on pharmacological properties of neferine-A bisbenzylisoquinoline alkaloid from *Nelumbo nucifera*. J. Food Biochem..

[11] Gokul P., Sathiyamurthy V.A., Padmapriya S., Kalaiyarasi R., Muthurajan R., Maduraimuthu D. (2025). Unveiling horticultural excellence of lotus (*Nelumbo nucifera*): A review. Plant Sci. Today.

[12] Guo H.B. (2008). Cultivation of lotus (*Nelumbo nucifera* Gaertn. ssp. *nucifera*) and its utilization in China. Genet. Resour. Crop Evol..

[13] Sun H., Li J.J., Song H.Y., Yang D., Deng X.B., Liu J., Wang Y.M., Ma J.Y., Xiong Y.Q., Liu Y.L. (2020). Comprehensive analysis of *AGPase* genes uncovers their potential roles in starch biosynthesis in lotus seed. BMC Plant Biol..

[14] Wang L., Fu J.L., Li M., Fragner L., Weckwerth W., Yang P.F. (2016). Metabolomic and proteomic profiles reveal the dynamics of primary metabolism during seed development of lotus (*Nelumbo nucifera*). Front. Plant Sci..

[15] Li J.J., Shi T., Huang L.Y., He D.L., Nyong’A T.M., Yang P.F. (2018). Systematic transcriptomic analysis provides insights into lotus (*Nelumbo nucifera*) seed development. Plant Growth Regul..

[16] Li J.J., Chen L., Song H.Y., Xin J., Li C.C., Yang M., Sun H. (2024). Systematic analysis of the expansin gene family in Nelumbo reveals candidate seed development responsive members in lotus (*Nelumbo nucifera*). Sci. Hortic..

[17] Li W., Qi L., Lin X.D., Chen H.H., Ma Z.Q., Wu K.Q., Huang S.Z. (2009). The expression of manganese superoxide dismutase gene from *Nelumbo nucifera* responds strongly to chilling and oxidative stresses. J. Integr. Plant Biol..

[18] Luo S.F., Hu H.L., Wang Y., Zhou H.S., Zhang Y.T., Zhang L.G., Li P.X. (2020). The role of melatonin in alleviating the postharvest browning of lotus seeds through energy metabolism and membrane lipid metabolism. Postharvest Biol. Technol..

[19] Punia Bangar S., Dunno K., Kumar M., Mostafa H., Maqsood S. (2022). A comprehensive review on lotus seeds (*Nelumbo nucifera* Gaertn.): Nutritional composition, health-related bioactive properties, and industrial applications. J. Funct. Foods.

[20] Song H.Y., Sun H., Dong G.Q., Yang H., Xin J., Yang D., Deng X.B., Liu J., Su Y.Y., Yang M. (2024). *NnSBE1* encodes a starch branching enzyme involved in starch biosynthesis in lotus seeds. Int. J. Biol. Macromol..

[21] Guo Z.B.B., Liu L., Zheng B.D. (2024). Chapter 12-lotus seed starch: Modification, structure, digestive properties, and probiotics. Non-Conventional Starch Sources.

[22] MacNeill G.J., Mehrpouyan S., Minow M.A.A., Patterson J.A., Tetlow I.J., Emes M.J. (2017). Starch as a source, starch as a sink: The bifunctional role of starch in carbon allocation. J. Exp. Bot..

[23] Huang L.C., Tan H.Y., Zhang C.Q., Li Q.F., Liu Q.Q. (2021). Starch biosynthesis in cereal endosperms: An updated review over the last decade. Plant Commun..

[24] Zhang D., Liu T., Sheng J., Lv S., Ren L. (2021). TMT-Based quantitative proteomic analysis reveals the physiological regulatory networks of embryo dehydration protection in lotus (*Nelumbo nucifera*). Front. Plant Sci..

[25] Zhu F.L., Sun H., Diao Y., Zheng X.W., Xie K.Q., Hu Z.L. (2019). Genetic diversity, functional properties and expression analysis of *NnSBE* genes involved in starch synthesis of lotus (*Nelumbo nucifera* Gaertn.). PeerJ.

[26] Song H.Y., Xin J., Yang D., Dong G.Q., Deng X.B., Liu J., Zhang M.H., Chen L., Su Y.Y., Yang H. (2024). *NnSUS1* encodes a sucrose synthase involved in sugar accumulation in lotus seed cotyledons. Plant Physiol. Biochem..

[27] Hagel J.M., Facchini P.J. (2013). Benzylisoquinoline alkaloid metabolism: A century of discovery and a brave new world. Plant Cell Physiol..

[28] Menéndez-Perdomo I.M., Facchini P.J. (2020). Isolation and characterization of two O-methyltransferases involved in benzylisoquinoline alkaloid biosynthesis in sacred lotus (*Nelumbo nucifera*). J. Biol. Chem..

[29] Pyne M.E., Gold N.D., Martin V.J.J. (2023). Pathway elucidation and microbial synthesis of proaporphine and bis-benzylisoquinoline alkaloids from sacred lotus (*Nelumbo nucifera*). Metab. Eng..

[30] Menéndez-Perdomo I.M., Facchini P.J. (2023). Elucidation of the (*R*)-enantiospecific benzylisoquinoline alkaloid biosynthetic pathways in sacred lotus (*Nelumbo nucifera*). Sci. Rep..

[31] Hao C.Y., Yu Y.T., Liu Y., Liu A., Chen S. (2024). The *CYP80A* and *CYP80G* are involved in the biosynthesis of benzylisoquinoline alkaloids in the sacred lotus (*Nelumbo nucifera*). Int. J. Mol. Sci..

[32] Sun H., Song H.Y., Deng X.B., Liu J., Yang D., Zhang M.H., Wang Y.X., Xin J., Chen L., Liu Y.L. (2022). Transcriptome-wide characterization of alkaloids and chlorophyll biosynthesis in lotus plumule. Front. Plant Sci..

[33] Zhao X., Zhao R., Yang X.J., Sun L.H., Bao Y.M., Liu Y.S., Blennow A., Liu X.X. (2023). Recent advances on bioactive compounds, biosynthesis mechanism, and physiological functions of *Nelumbo nucifera*. Food Chem..

[34] Yu Y.T., Wei X.L., Liu Y., Dong G.Q., Hao C.Y., Zhang J., Jiang J.Z., Cheng J.T., Liu A., Chen S. (2022). Identification and quantification of oligomeric proanthocyanidins, alkaloids, and flavonoids in lotus seeds: A potentially rich source of bioactive compounds. Food Chem..

[35] Chen S., Fang L.C., Xi H.F., Guan L., Fang J.B., Liu Y.L., Wu B.H., Li S.H. (2012). Simultaneous qualitative assessment and quantitative analysis of flavonoids in various tissues of lotus (*Nelumbo nucifera*) using high performance liquid chromatography coupled with triple quad mass spectrometry. Anal. Chim. Acta.

[36] Pei H.T., Su W.Y., Gui M., Dou M.J., Zhang Y.X., Wang C.Z., Lu D. (2021). Comparative analysis of chemical constituents in different parts of lotus by UPLC and QToF-MS. Molecules.

[37] Wu L., Xiong W., Hu J.W., Gu Z., Xu J.G., Si C.L., Bae Y.S., Xu G. (2018). Purification of four flavonoid glycosides from lotus (*Nelumbo nucifera* Gaertn) plumule by macroporous resin combined with HSCCC. J. Chromatogr. Sci..

[38] Li S.S., Wu J., Chen L.G., Du H., Xu Y.J., Wang L.J., Zhang H.J., Zheng X.C., Wang L.S. (2014). Biogenesis of *C*-glycosyl flavones and profiling of flavonoid glycosides in lotus (*Nelumbo nucifera*). PLoS ONE.

[39] Liu T., Zhu M.Z., Zhang C.Y., Guo M.Q. (2017). Quantitative analysis and comparison of flavonoids in lotus plumules of four representative lotus cultivars. J. Spectro..

[40] Feng C.Y., Li S.S., Yin D.D., Zhang H.J., Tian D.K., Wu Q., Wang L.J., Su S., Wang L.S. (2016). Rapid determination of flavonoids in plumules of sacred lotus cultivars and assessment of their antioxidant activities. Ind. Crops Prod..

[41] Zheng J.X., Tian W.Y., Yang C., Shi W.P., Cao P.H., Long J.T., Xiao L.M., Wu Y., Liang J.Z., Li X.B. (2019). Identification of flavonoids in plumula nelumbinis and evaluation of their antioxidant properties from different habitats. Ind. Crops Prod..

[42] Zhu M.Z., Liu T., Zhang C.Y., Guo M.Q. (2017). Flavonoids of lotus (*Nelumbo nucifera*) seed embryos and their antioxidant potential. J. Food Sci..

[43] Peng Y., Gan R.Y., Li H.B., Yang M.X., McClements D.J., Gao R.C., Sun Q.C. (2021). Absorption, metabolism, and bioactivity of vitexin: Recent advances in understanding the efficacy of an important nutraceutical. Crit. Rev. Food Sci. Nutr..

[44] Feng C.Y., Li S.S., Taguchi G., Wu Q., Yin D.D., Gu Z.Y., Wu J., Xu W.Z., Liu C., Wang L.S. (2021). Enzymatic basis for stepwise *C*-glycosylation in the formation of flavonoid di-C-glycosides in sacred lotus (*Nelumbo nucifera* Gaertn.). Plant J..

[45] Luo S.F., Hu H.L., Zhang L.G., Zhou H.S., Li P.X. (2017). Sugars in postharvest lotus seeds were modified by 6-benzylaminopurine treatment through altering related enzymes involved in starch-sucrose metabolism. Sci. Hortic..

[46] Tu Y.X., Yan S.L., Li J. (2020). Impact of harvesting time on the chemical composition and quality of fresh lotus seeds. Hortic. Environ. Biotechnol..

[47] Yang Y.A., Liu R.L., Han Y.C., Wu W.J., Fang X.J., Mu H.L., Gao H.Y., Chen H.J. (2023). Critical taste substances and regulatory pathways of fresh lotus seed pulps at different ripeness stages. Postharvest Biol. Technol..

[48] Tilley A., McHenry M.P., McHenry J.A., Solah V., Bayliss K. (2023). Enzymatic browning: The role of substrates in polyphenol oxidase mediated browning. Curr. Res. Food Sci..

[49] Cai X.X., Hong Y.X., Wang S.Y., Zhao L.N., Rao P.F. (2015). Purification and enzymatic characteristics of a novel polyphenol oxidase from lotus seed (*Nelumbo nucifera* Gaertn.). Int. J. Food Sci. Technol..

[50] Chen L., Song H.Y., Dong G.Q., Xin J., Yang M., Su Y.Y., Sun H. (2026). Physiological and molecular mechanisms underlying the deterioration of external quality in postharvest fresh lotus seed. Postharvest Biol. Technol..

[51] Chen L., Dong G.Q., Song H.Y., Xin J., Su Y.Y., Cheng W., Yang M., Sun H. (2024). Unveiling the molecular dynamics of low temperature preservation in postharvest lotus seeds: A transcriptomic perspective. BMC Plant Biol..

[52] Xu T., Chen Y., Kang H. (2019). Melatonin is a potential target for improving post-harvest preservation of fruits and vegetables. Front. Plant Sci..

[53] Min D.D., Li F.J., Zhang X.H., Shu P., Cui X.X., Dong L.L., Ren C.T., Meng D.M., Li J. (2018). Effect of methyl salicylate in combination with 1-methylcyclopropene on postharvest quality and decay caused by botrytis cinerea in tomato fruit. J. Sci. Food Agric..

[54] Li P.X., Gao J.X., Hu H.L., Luo S.F., Zhang L.G. (2016). Postharvest senescence of fresh lotus pods and seeds is delayed by treatment with 1-methylcyclopropene. Ann. Appl. Biol..

[55] Li P.X., Hu H.L., Luo S.F., Zhang L.G., Gao J.X. (2017). Shelf life extension of fresh lotus pods and seeds (*Nelumbo nucifera* Gaertn.) in response to treatments with 1-MCP and lacquer wax. Postharvest Biol. Technol..

[56] Chen S.N., Xie R.P., Li J., Fan Y.W., Liu X.R., Zhang B., Deng Z.Y. (2018). Alteration on phenolic acids and the appearance of lotus (*Nelumbo nucifera* Gaertn) seeds dealt with antistaling agents during storage. Int. J. Food Prop..

[57] Sun L., Luo S.F., Hu H.L., Zhou H.S., Zhang Y.T., An R.H., Ling J., Li P.X. (2022). Melatonin promotes the normal cellular mitochondrial function of lotus seeds through stimulating nitric oxide production. Postharvest Biol. Technol..

[58] Xu C.F., Wang W., Yan S.L., Li J. (2023). A trigger non-contact antibacterial packaging based on modified basic magnesium hypochlorite (BMH) and application on preservation effect of fresh lotus seeds. Food Packag. Shelf Life.

[59] Chi Y.Y., Luo L.P., Huang X.Y., Cui M., Dai X.M., Hao Y.B., Guo X.L., Luo H.L. (2019). Rapid determination of the freshness of lotus seeds using surface desorption atmospheric pressure chemical ionization-mass spectrometry with multivariate analyses. J. Food Qual..

[60] Salvi P., Varshney V., Majee M. (2022). Raffinose family oligosaccharides (RFOs): Role in seed vigor and longevity. Biosci. Rep..

[61] Liang W.Z., Dong H.X., Guo X.J., Rodríguez V., Cheng M.P., Li M.L., Benech-Arnold R., Pu Z.E., Wang J.R. (2023). Identification of long-lived and stable mRNAs in the aged seeds of wheat. Seed Biol..

[62] Shen-Miller J., Schopf J.W., Harbottle G., Cao R.J., Ouyang S., Zhou K.S., Southon J.R., Liu G.H. (2002). Long-living lotus: Germination and soil gamma-irradiation of centuries-old fruits, and cultivation, growth, and phenotypic abnormalities of offspring. Am. J. Bot..

[63] Shen-Miller J., Aung L.H., Turek J., Schopf J.W., Tholandi M., Yang M., Czaja A. (2013). Centuries-old viable fruit of sacred lotus *Nelumbo nucifera* Gaertn var. China Antique. Trop Plant Biol..

[64] Sun H., Xin J., Ullah A., Song H.Y., Chen L., Yang D., Deng X.B., Liu J., Ming R., Zhang M.H. (2025). Unveiling the secrets of lotus seed longevity: Insights into adaptive strategies for extended storage. J. Exp. Bot..

[65] Shen-Miller J., Lindner P., Xie Y.M., Villa S., Wooding K., Clarke S.G., Loo R.R., Loo J.A. (2013). Thermal-stable proteins of fruit of long-living sacred lotus *Nelumbo nucifera* Gaertn var. China Antique. Trop. Plant Biol..

[66] Ding Y., Cheng H.Y., Song S.Q. (2008). Changes in extreme high-temperature tolerance and activities of antioxidant enzymes of sacred lotus seeds. Sci. China C Life Sci..

[67] Chu P., Chen H.H., Zhou Y.L., Li Y., Ding Y., Jiang L.W., Tsang E.W., Wu K., Huang S.Z. (2012). Proteomic and functional analyses of *Nelumbo nucifera* annexins involved in seed thermotolerance and germination vigor. Planta.

[68] Dong C., Zheng X.F., Diao Y., Wang Y.W., Zhou M.Q., Hu Z.L. (2015). Molecular Cloning and Expression analysis of a catalase gene (*NnCAT*) from *Nelumbo nucifera*. Appl. Biochem. Biotechnol..

[69] Zhou Y.L., Chen H.H., Chu P., Li Y., Tan B., Ding Y., Tsang E.W., Jiang L., Wu K., Huang S.Z. (2012). *NnHSP17.5*, a cytosolic class II small heat shock protein gene from *Nelumbo nucifera*, contributes to seed germination vigor and seedling thermotolerance in transgenic *Arabidopsis*. Plant Cell Rep..

[70] Zhou Y.L., Chu P., Chen H.H., Li Y., Liu J., Ding Y., Tsang E.W., Jiang L., Wu K., Huang S.Z. (2012). Overexpression of *Nelumbo nucifera* metallothioneins 2a and 3 enhances seed germination vigor in *Arabidopsis*. Planta.

[71] Chen H.H., Chu P., Zhou Y.L., Ding Y., Li Y., Liu J., Jiang L.W., Huang S.Z. (2016). Ectopic expression of *NnPER1*, a *Nelumbo nucifera* 1-cysteine peroxiredoxin antioxidant, enhances seed longevity and stress tolerance in *Arabidopsis*. Plant J..

[72] Ming R., VanBuren R., Liu Y.L., Yang M., Han Y.P., Li L.T., Zhang Q., Kim M.J., Schatz M.C., Campbell M. (2013). Genome of the long-living sacred lotus (*Nelumbo nucifera* Gaertn.). Genome Biol..

[73] Hu J.H., Jin J., Qian Q., Huang K.K., Ding Y. (2016). Small RNA and degradome profiling reveals miRNA regulation in the seed germination of ancient eudicot *Nelumbo nucifera*. BMC Genomics.

[74] Sharma B.R., Gautam L.N., Adhikari D., Karki R.A. (2017). Comprehensive review on chemical profiling of *Nelumbo nucifera*: Potential for drug development. Phytother. Res..

[75] Zhou M.G., Jiang M., Ying X.H., Cui Q.X., Han Y.Q., Hou Y.Y., Gao J., Bai G., Luo G.A. (2013). Identification and comparison of anti-inflammatory ingredients from different organs of Lotus *Nelumbo* by UPLC/Q-TOF and PCA coupled with a NF-κB reporter gene assay. PLoS ONE.

[76] Fang Y.T., Li Q., Shao Q., Wang B.H., Wei Y. (2017). A general ionic liquid pH-zone-refining countercurrent chromatography method for separation of alkaloids from *Nelumbo nucifera* Gaertn. J. Chromatogr. A.

[77] Kashiwada Y., Aoshima A., Ikeshiro Y., Chen Y.P., Furukawa H., Itoigawa M., Fujioka T., Mihashi K., Cosentino L.M., Morris-Natschke S.L. (2005). Anti-HIV benzylisoquinoline alkaloids and flavonoids from the leaves of *Nelumbo nucifera*, and structure-activity correlations with related alkaloids. Bioorg. Med. Chem..

[78] Nishimura K., Horii S., Tanahashi T., Sugimoto Y., Yamada J. (2013). Synthesis and pharmacological activity of alkaloids from embryo of lotus, *Nelumbo nucifera*. Chem. Pharm. Bull..

[79] Itoh A., Saitoh T., Tani K., Uchigaki M., Sugimoto Y., Yamada J., Nakajima H., Ohshiro H., Sun S., Tanahashi T. (2011). Bisbenzylisoquinoline Alkaloids from *Nelumbo nucifera*. Chem. Pharm. Bull..

[80] Qian J.Q. (2002). Cardiovascular pharmacological effects of bisbenzylisoquinoline alkaloid derivatives. Acta Pharmacol. Sin..

[81] Zhu M.Z., Liu T., Guo M.Q. (2016). Current advances in the metabolomics study on lotus seeds. Front. Plant Sci..

[82] Wang Q., Zheng Y.F., Zhuang W.J., Lu X., Luo X.L., Zheng B.D. (2018). Genome-wide transcriptional changes in type 2 diabetic mice supplemented with lotus seed resistant starch. Food Chem..

[83] Zeng H.L., Zheng Y.X., Lin Y., Huang C.C., Lin S., Zheng B.D., Zhang Y. (2018). Effect of fractionated lotus seed resistant starch on proliferation of *Bifidobacterium longum* and *Lactobacillus delbrueckii* subsp. *bulgaricus* and its structural changes following fermentation. Food Chem..

[84] Cao Y., Kou R.B., Huang X.Y., Wang N.L., Di D.L., Wang H., Liu J.F. (2024). Separation of polysaccharides from *Lycium barbarum* L. by high-speed countercurrent chromatography with aqueous two-phase system. Int. J. Biol. Macromol..

[85] Wu J.W., Ge F.H., Wei D.M., Xu X.J. (2016). Combination of supercritical fluid extraction with high-speed countercurrent chromatography for extraction and isolation of ethyl p-methoxycinnamate and ethyl cinnamate from *Kaempferia galanga* L.. Sep. Sci. Technol..

[86] Naake T., Zhu F., Alseekh S., Scossa F., Perez de Souza L., Borghi M., Brotman Y., Mori T., Nakabayashi R., Tohge T. (2024). Genome-wide association studies identify loci controlling specialized seed metabolites in *Arabidopsis*. Plant Physiol..

[87] Zhang M.Y., Kong D.X., Wang H.Y. (2023). Genomic landscape of maize domestication and breeding improvement. Seed Biol..

[88] Liang S., Duan Z.B., He X.M., Yang X., Yuan Y.Q., Liang Q.J., Pan Y., Zhou G.A., Zhang M., Liu S.L. (2024). Natural variation in *GmSW17* controls seed size in soybean. Nat. Commun..

[89] Jiang Y.F., Zhou M.G., Ke S.M., Deng X.X., Li Y.S. (2024). *GSW3.1*, a novel gene controlling grain size and weight in rice. Crop J..

[90] Li J.K., Huang S.Q. (2009). Effective pollinators of Asian sacred lotus (*Nelumbo nucifera*): Contemporary pollinators may not reflect the historical pollination syndrome. Ann. Bot..

[91] Grant N., Miller R., Watling J., Robinson S. (2010). Distribution of thermogenic activity in floral tissues of *Nelumbo nucifera*. Funct. Plant Biol..

[92] Zheng P., Sun H., Liu J., Lin J.S., Zhang X.T., Qin Y., Zhang W.P., Xu X.M., Deng X.B., Yang D. (2022). Comparative analyses of American and Asian lotus genomes reveal insights into petal color, carpel thermogenesis and domestication. Plant J..

[93] Magar B.T., Acharya S., Gyawali B., Timilsena K., Upadhayaya J., Shrestha J. (2021). Genetic variability and trait association in maize (*Zea mays* L.) varieties for growth and yield traits. Heliyon.

[94] Carr D.E., Dudash M.R. (2003). Recent approaches into the genetic basis of inbreeding depression in plants. Philos. Trans. R. Soc. Lond. B Biol. Sci..

[95] Bareke T., Addi A. (2019). Effect of honeybee pollination on seed and fruit yield of agricultural crops in *Ethiopia*. MOJ Ecol. Environ. Sci..

[96] Khatfan A., Li Z., Chen L.Q., Riablershirun N., Sathornviriyapong V., Juntawong N. (2014). Pollen viability, germination, and seed setting of *Nelumbo nucifera*. ScienceAsia.

[97] Ma Y.P., Devi M.J., Song L.H., Gao H.D., Jin L., Cao B. (2024). Multi-omics integration analysis reveals metabolic regulation of carbohydrate and secondary metabolites during goji berry (*Lycium barbarum* L.) maturation. Postharvest Biol. Technol..

[98] Ma L.L., Zheng Y.Y., Sang Z.Z., Ge Y.H., Bai C.M., Fu A.Z., Wang Q., Watkins C.B., Zuo J.H. (2023). Multi-omics analysis reveals the mechanism of calcium-reduced quality deterioration in mechanically injured green pepper fruit. Postharvest Biol. Technol..

[99] Qu B., Shao G.Q., Yang N., Pan K., Xiao Z.L., Luo Y.C. (2024). Revolutionizing food sustainability: Leveraging magnetic fields for food processing and preservation. Trends Food Sci. Technol..

[100] Wang D.Y., Zhang M., Li M., Lin J.C. (2024). Fruits and vegetables preservation based on AI technology: Research progress and application prospects. Comput. Electron. Agric..

[101] Chen T., Ji D.C., Zhang Z.Q., Li B.Q., Qin G.Z., Tian S.P. (2021). Advances and strategies for controlling the quality and safety of postharvest fruit. Engineering.

[102] Yan C., Kim S.R., Ruiz D.R., Farmer J.R. (2022). Microencapsulation for food applications: A review. ACS Appl. Bio Mater..

[103] Liu K.J., Xing S.H., Abd El-Aty A.M., Tan M.Q. (2025). Precision nutrition based on food bioactive components assisted by delivery nanocarriers for ocular health. Trends Food Sci. Technol..

[104] Mallela V.J., Rudrapal M., Prasanth D., Pasala P.K., Bendale A.R., Bhattacharya S., Aldosari S.M., Khan J. (2024). Lotus seed (*Nelumbinis semen*) extract: Anticancer potential and chemoprofiling by in vitro, in silico and GC-MS studies. Front Chem..

[105] Zhang L.Y., Zhang M., Mujumdar A.S., Chen Y.P. (2024). From farm to market: Research progress and application prospects of artificial intelligence in the frozen fruits and vegetables supply chain. Trends Food Sci. Technol..

