# Supplementary material for: Lotus Seeds: Current Molecular Biology Insights and Future Perspectives as a Prominent Biological Resource
Source: Plants (Basel). 2026 Jan 2;15(1):136. doi: 10.3390/plants15010136 (PMC12788056; doi:10.3390/plants15010136)
Supplement: Supplementary file 1 [file plants-15-00136-s001.zip › plants-4043403-supplementary.pdf]

Table S1 Primary bioactive components of lotus seeds and their functions

Compound	Funtions	References
**Bisbenzylisoquinoline**		
Neferine	Antioxidant, anti-inflammatory, anti-proliferative, anti-cancer, anti-cardiovascular, etc.	[2,74,75]
Liensinine	Anti-HIV, anti-cardiovascular, anti-cancer, etc.	[76,77]
Isoliensinine	Antioxidant, anti-inflammatory, anti-proliferative, anti-cancer, anti-cardiovascular, anti-tumour, etc.	[74,77]
N-norisoliensinine	Anti-cardiovascular, anti-tumour, antioxidant, anti-inflammatory, etc.	[74]
Methyl neferine	Anti-cardiovascular, anti-cardiovascular, anti-tumour, etc.	[78]
Nelumboferine	Lower blood pressure, anti-cardiovascular, etc.	[78,79]
Negferine	Anti-HIV, anti-inflammatory, anti-cardiovascular, etc.	[74,77]
Nelumborine	Anti-proliferative, anti-cardiovascular, anti-diabetic, etc.	[78]
Dauricine	Anti-HIV, anti-cardiovascular, antibacterial, lower blood pressure, etc.	[74,80]
**Flavonoids**		
Schaftoside	Antioxidant, anti-inflammatory, anti-cardiovascular, neuroprotection, etc.	[38,81]
Isoschaftoside	Antioxidant, anti-inflammatory, neuroprotection, lower blood sugar, etc.	[38]
Vitexin	Antioxidant, anti-inflammatory, anti-proliferative, anti-tumour, anti-cardiovascular, etc.	[38]
Isovitexin	Antioxidant, anti-inflammatory, neuroprotection, lower blood sugar, etc.	[38,81]
Orientin	Antioxidant, anti-inflammatory, neuroprotection, etc.	[38]
Isoorientin	Antioxidant, anti-inflammatory, neuroprotection, lower blood sugar, etc.	[38]
Apigenin-6,8-di-*C*-glucose	Antioxidant, anti-inflammatory, etc.	[38]
Luteolin-6-*C*-glucose-8-*C*-pentose	Antioxidant, anti-inflammatory, etc.	[38]
Luteolin-6-*C*-pentose-8-*C*-glucose	Antioxidant, anti-inflammatory, etc.	[38]
Apigenin-6-*C*-glucose-8-*C*-xylose	Antioxidant, anti-inflammatory, etc.	[38]
Apigenin-6-*C*-xylose-8-*C*-glucose	Antioxidant, anti-inflammatory, etc.	[38]
Apigenin-6-*C*-glucose-8-*C*-rhamnose	Antioxidant, anti-inflammatory, etc.	[38]
Apigenin-6-*C*-rhamnose-8-*C*-glucose	Antioxidant, anti-inflammatory, etc.	[38,81]
Rutin	Antioxidant, anti-inflammatory, anti-cancer, lower blood sugar, etc.	[35,81]
Hyperoside	Antioxidant, anti-inflammatory, anti-cancer, anti-cardiovascular, etc.	[35,81]
Isoquercitrin	Antioxidant, anti-inflammatory, anti-cancer, anti-	[35,81]

	proliferative, and lower blood sugar, etc.	
Quercetin	Antioxidant, anti-inflammatory, anti-cancer, anti-proliferative, lower blood sugar, etc.	[35,81]
Kaempferol 3-*O*-robinobioside	Antioxidant, anti-inflammatory, etc.	[35,81]
Isorhamnetin 3-*O*-rutinoside	Antioxidant, anti-inflammatory, anti-cancer, etc.	[35,81]
Isorhamnetin-3-neohesperidose	Antioxidant, lower blood lipids, etc.	[38]
Diosmetin-7-rutinose	Antioxidant, anti-inflammatory, etc.	[38]
Quercetin-3-neohesperidose	Antioxidant, anti-inflammatory, etc.	[38,81]
Luteolin-7-rutinose	Antioxidant, anti-inflammatory, neuroprotection, etc.	[38,81]
Myricetin 3-*O*-glucoside	Antioxidant, anti-inflammatory, anti-tumour, etc.	[35,81]
Myricetin 3-*O*-glucuronide	Antioxidant, anti-inflammatory, etc.	[35,81]
Quercetin 3-*O*-glucuronide	Antioxidant, anti-inflammatory, liver protection, etc.	[35,81]
Astragalin	Antioxidant, anti-inflammatory, lower blood sugar, anti-tumour, etc.	[35,81]
Syringetin 3-*O*-glucoside	Antioxidant, anti-inflammatory, neuroprotection, etc.	[35,81]
Isorhamnetin 3-*O*-glucoside	Antioxidant, anti-inflammatory, lower blood sugar, anti-tumour, etc.	[35,81]
Kaempferol 3-*O*-glucuronide	Antioxidant, anti-inflammatory, anti-tumour, etc.	[35,81]

Table S2 Genes involved in lotus seed development and their functions

Gene	Gene id	Gene Description	Functions	Part	References
**Starch and sucrose metabolism**					
NnEXPA2	Nn2g10323	Expansin gene family	Enhancing cell expansion during the early development.	Cotyledon	[16]
NnSUS1	Nn2g14684	Sucrose synthase	Increase total soluble sugar levels.	Cotyledon	[26]
NnSS	Nn4g23188	Soluble starch synthase	starch biosynthesis	Cotyledon	[64]
NnSWEET14	Nn5g28575	Sugar transport-related SWEET gene family	Increase soluble sugar content	Cotyledon	[3]
NnAGPL2a	Nn5g29669	ADP-glucose pyrophosphorylase	Involved in starch biosynthesis	Cotyledon	[13]
NnAGPS1a	Nn3g18870	ADP-glucose pyrophosphorylase	Involved in starch biosynthesis	Cotyledon	[13]
NnSBE1	Nn5g30949	Starch branching enzyme	Involved in amylopectin and total starch biosynthesis	Cotyledon	[20]
NnGBSS1	Nn1g00434	Granule-bound starch syntha se	Involved in starch biosynthesis	Cotyledon	[64]
NnSUS4	Nn1g00434	Sucrose synthase	Involved in cotyledon starch biosynthesis	Cotyledon	[64]
NnSPSB	Nn1g07936	Starch branching enzyme	Involved in cotyledon starch biosynthesis	Cotyledon	[64]
NnPHS2	Nn2g11360	Pre-harvest sprouting gene	Involved in cotyledon starch biosynthesis	Cotyledon	[64]
NnDPE1	Nn1g05090	Disproportionating Enzymes	Involved in cotyledon starch biosynthesis	Cotyledon	[64]
**Alkaloid biosynthesis**					
NnTYDC	NNU_22559	Tyrosine decarboxylase	Involved in bis-BIAs biosynthetic	Plumule	[32]
NnCNMT	NNU_11880	*Catechol-O-methyltransferase*	Involved in bis-BIAs biosynthetic	Plumule	[32]
NnOMTs	NNU_15801, NNU_15809, NNU_25948	*O*-methylation	Catalyzes the O-methyltransferase (OMT) transfer of a methyl group to a hydroxyl group of an alkaloid substrate	Plumule	[32]

NnNCS	NNU_14334	Norcoclaurine synthase	Involved in bis-BIAs biosynthetic	Plumule	[32]
Nn6OMTs	NNU_19035, NNU_03166, etc.	6-*O*-methyltransferase	Involved in bis-BIAs biosynthetic	Plumule	[32]
Nn7OMTs	NNU_04966, NNU_04906, etc.	7-*O*-methyltransferase	Involved in bis-BIAs biosynthetic	Plumule	[32]
NnCYP80A	NNU_21373	*Cytochrome P450* gene	Catalyze C-O coupling in (*R*)-N-methylcoclaurine, involved in bis-BIAs biosynthetic	Plumule	[8,31]
**Chlorophyll (Chl) biosynthesis**					
NnGSA	NNU_22236 Nn4g22230	Chl biosynthesis genes	Involved in Chl biosynthesis	Plumule	[32,64]
NnHEME1	NNU_12265	Chl biosynthesis genes	Involved in Chl biosynthesis	Plumule	[32]
NnCHLG1	NNU_12622	Chl biosynthesis genes	Involved in Chl biosynthesis	Plumule	[32]
NnPOR1	NNU_01188	Chl biosynthesis genes	Involved in Chl biosynthesis	Plumule	[32]
NnCHLH1	NNU_03121 Nn7g36281	Chl biosynthesis genes	Involved in Chl biosynthesis	Plumule	[32,64]
NnPOR2	NNU_16195	Chl biosynthesis genes	Involved in Chl biosynthesis	Plumule	[32]
NnHEMG	NNU_02121	Chl biosynthesis genes	Involved in Chl biosynthesis	Plumule	[32]
NnDVR	NNU_06919	Chl biosynthesis genes	Involved in Chl biosynthesis	Plumule	[32]
NnCAOs	NNU_24327, NNU_19596, Nn1g08791	Chl biosynthesis genes	Involved in Chl biosynthesis	Plumule	[32,64]
NnHEMC	NNU_01206	Chl biosynthesis genes	Involved in Chl biosynthesis	Plumule	[32]
NnCRD	NNU_10837	Chl biosynthesis genes	Involved in Chl biosynthesis	Plumule	[32]
NnHEMB	NNU_04375	Chl biosynthesis genes	Involved in Chl biosynthesis	Plumule	[32]
**Flavonoid biosynthesis**					
NnCGT1/2	UGT708N1, UGT708N2	*C-glycosyltransferase* (*CGT*) genes	Involved in flavonoid di-*C*-glycosides biosynthesis	Plumule	[44]

NnANS	Nn1g01275	Leucoanthocyanidin dioxygenase/anthocyanidin synthase	Associated with flavonoids biosynthesis	Pericarp	[64]
NnCHS	Nn3g19912	Chalcone synthase	Associated with flavonoids biosynthesis	Pericarp	[64]
**Pericarp morphogenesis**					
NnCesAs	Nn8g39978, Nn8g40892, etc.	Cellulose synthase	Cellulose synthesis in the pericarp cell wall	Pericarp	[64]
NnCERs	Nn4g26388, Nn1g01246, etc.	Eceriferum gene family	Involved in pericarp wax biosynthesis	Pericarp	[64]
NnKCS6	Nn3g17828	3-ketoacyl-CoA synthase	Involved in pericarp wax biosynthesis	Pericarp	[64]
NnMYB96	Nn4g23160	MYB gene family	Involved in pericarp wax biosynthesis	pericarp	[64]
NnCADs	Nn5g30425	*Coronary artery disease* genes	Involved in cell wall lignin biosynthesis	Pericarp	[64]
**Seed longevity**					
NnPER1	Nn3g21870	1-*cys* peroxiredoxin gene	Scavenges excess reactive oxygen species (ROS) to protect seeds from oxidative stress damage	Plumule	[71]
NnLEAs	Nn4g26333, Nn1g01694, etc.	Late embryogenesis abundant (LEA) proteins	Improving seed storage tolerance in response to dehydration stress during late seed development	Plumule; Cotyledon	[1]
NnHSP17.5	EF100453.1	Cytosolic class II small heat shock protein gene	Involved in oxidative stress responses to enhancing seed tolerance	Plumule	[69]
CuZn-SOD	NNU_001676	Copper/zinc superoxide dismutase	Maintains seed viability and safeguards cell integrity under high temperature treatment	Plumule; Cotyledon	[65]
HSP80	NNU_010290	Heat shock proteins	Enhance seed heat resistance, providing protection and repair under stress	Plumule; Cotyledon	[65]
PIMT	NNU_002938, NNU_004234	L-isoleucyl-O-methyltransferase	Protein-repair activity after heating to 100°C	Plumule; Cotyledon	[65]

NnMT2a */MT3*	EF421200, EF42120, EF421199	*Metallothionein* genes	Enhance seed resistance to stresses.	Plumule	[70]
NnANN1	——	*Annexin* genes	Protects the membrane from peroxidation and regulates heat resistance and germination of seeds.	Plumule	[67]
NnCIPK	Nn3g20370	water deficit response gene	Responding to dehydration stress	Plumule	[64]
NnDERB	Nn2g11006	ABA response genes	confers tolerance to various abiotic stresses	Plumule	[64]
NnPYL4	Nn1g09182	ABA response genes	Protect the lotus seed plumule against dehydration stress	Plumule	[64]
NnPYL8	Nn3g19033	ABA response genes	Protect the lotus seed plumule against dehydration stress	Plumule	[64]
NnPYR1	Nn6g33591	ABA response genes	Protect the lotus seed plumule against dehydration stress	Plumule	[64]
NnABF2	Nn5g30287	Basic leucine zipper protein	Regulates abscisic acid (ABA)-dependent stress-responsive gene expression	Plumule	[64]
NnABI4	Nn7g38338	*Abscisic acid insensitive 4* gene	Protect the lotus seed plumule against dehydration stress	Plumule	[64]
NnHSP17.6C	Nn3g20991	Heat shock proteins	Mitigate the detrimental effects of dehydration during their development	Plumule	[64]
NnHSP70	Nn8g39973	Heat shock proteins	Mitigate the detrimental effects of dehydration during their development	Plumule	[64]
NnHSP17.8	Nn5g28904	Heat shock proteins	Mitigate the detrimental effects of dehydration during their development	Plumule	[64]
NnHSP89.1	Nn2g10218	Heat shock proteins	Mitigate the detrimental effects of dehydration during their development	Plumule	[64]

NnNYC1	Nn2g11727	Chl retention genes	Involved in Chl retention	Plumule	[64]
NnNOL	Nn2g14524	Chl retention genes	Involved in Chl retention	Plumule	[64]
NnPPH	Nn7g36359	Chl retention genes	Involved in Chl retention	Plumule	[64]
NnSGR1	Nn1g06329	Chl retention genes	A crucial regulator of Chl degradation results in a strong stay-green plant phenotype	Plumule	[64]
NnHSPs	Nn7g37861, Nn7g36887, etc.	Heat shock proteins	Down-regulation of *HSP* genes might have led to altered protein folding and processing, resulting in decreased viability of aging lotus seeds	Plumule	[64]
NnPGIP	Nn2g15554, Nn2g15567, etc.	Polygalacturonase inhibitor protein	Involved in plant defense against pathogens	——	[64]
NnCRAs	Nn3g19659, Nn1g07603, etc.	12S seed storage protein	Important for seed germination and early seedling growth	——	[64]

Table S3 Abbreviations of proper nouns

Number	Abbreviation	Full English name
1	DAP	Days after pollination
2	LSS	Lotus seed starch
3	UDP-glucose	Uridine diphosphate glucose
4	INV	Invertase
5	FK	Fructokinase
6	HK	Hexokinase
7	Glc6P	Glucose-6-phosphate
8	ADPGlc	ADP-glucose
9	AGPase	ADP-glucose pyrophosphorylase
10	GBSS	Granule-Bound Starch Synthase
11	SBE	Starch Branching Enzyme
12	SS	Soluble Starch Synthase
13	SuSy	Sucrose Synthase
14	SWEET	Sugars Will Eventually be Exported Transporter
15	Glc	Glucosehate
16	Fru	Fructose
17	Glc1P	Glucose 1-phosphate
18	UDPGlc	UDP-glucose
19	Fru6P	Fructose 6-phosp
20	PGI	Plastidial Phosphoglucose Isomerase
21	PGM	Plastidial Phosphoglucomutase
22	GPT	Glucose-Phosphate Transporter
23	BIAs	Benzylisoquinoline alkaloids
24	Bis-BIAs	Bis-benzylisoquinoline alkaloids
25	TYDC	Tyrosine Decarboxylase
26	NCS	Norcoclaurine Synthase
27	6OMT	Norcoclaurine 6-O-Methyltransferase
28	CNMT	Coclaurine N-Methyltransferase
29	CYP80A	(*S*)-N-Methylcoclaurine 3’-hydroxylase
30	7OMT	7-O-Methyltransferase
31	4’OMT	4’-O-Methyltransferase
32	HPLC-DAD	High-performance liquid chromatography with diode array detector
33	UPLC-ESI-QTOF-MS	Ultra-performance liquid chromatography with tandem mass spectrometry
34	LC-UV/LC-MS	Liquid chromatography with ultraviolet detection/liquid chromatography-mass spectrometry
35	PAL	Phenylalanine Ammonia Lyase
36	C4H	Cinnamate 4-Hydroxylase
37	4CL	4-Coumaryol CoA Ligase
38	CHS	Chalcone Synthase;
39	CHI	Chalcone Isomerise

40	F3H	Flavanone 3-hydroxylase
41	FLS	Flavonol synthase
42	F3'H	Flavonoid 3'-hydroxylase
43	UF3GaT	UDP-galactose: flavonoid 3-O-galactosyltransferase
44	GT	Glucosyltransferase
45	RT	Rhamnosyltransferase
46	F2H	Flavanone 2-Hydroxylase
47	CGT	Cyclodextrin glycosyltransferase
48	FNS	Flavone Synthase
49	UF7GT	UDP-glucose: flavanone 7-O-glucosyltransferase
50	FRK	Fructokinase
51	PEPC	Phosphoenolpyruvate carboxylase
52	NAD-MDH	NAD-malate dehydrogenase
53	WGCNA	Weighted gene co-expression network analysis
54	DEGs	Differentially expressed genes
55	1-MCP	1-Methylcyclopropene
56	AA	Ascorbic acid
57	BA	Benzoic acid
58	SDS	Sodium sulfite
59	6-BA	6-benzylaminopurine
60	MT	Melatonin
61	NO	Nitric oxide
62	DAPCI-MS	Desorption atmospheric pressure chemical ionization-mass spectrometry
63	BP	Before the present
64	SOD	Superoxide dismutase
65	CAT	Catalase
66	PER	Antioxidant 1-cys peroxidase
67	low-GI	Low-Glycemic Index
68	SFE	Supercritical fluid extraction
69	HSCCC	High-speed countercurrent chromatography
70	GWAS	Genome-wide association studies
71	QTL	Quantitative trait locus
